# Minimal Window Duration for Accurate HRV Recording in Athletes

**DOI:** 10.3389/fnins.2017.00456

**Published:** 2017-08-10

**Authors:** Nicolas Bourdillon, Laurent Schmitt, Sasan Yazdani, Jean-Marc Vesin, Grégoire P. Millet

**Affiliations:** ^1^Faculty of Biology and Medicine, Institute of Sport Sciences, University of Lausanne Lausanne, Switzerland; ^2^National Centre of Nordic-Ski, Research and Performance Premanon, France; ^3^Applied Signal Processing Group, Ecole Polytechnique Fédérale de Lausanne Lausanne, Switzerland

**Keywords:** accuracy, HRV, fatigue, RMSSD, LF-HF, athlete

## Abstract

Heart rate variability (HRV) is non-invasive and commonly used for monitoring responses to training loads, fitness, or overreaching in athletes. Yet, the recording duration for a series of RR-intervals varies from 1 to 15 min in the literature. The aim of the present work was to assess the minimum record duration to obtain reliable HRV results. RR-intervals from 159 orthostatic tests (7 min supine, SU, followed by 6 min standing, ST) were analyzed. Reference windows were 4 min in SU (min 3–7) and 4 min in ST (min 9–13). Those windows were subsequently divided and the analyses were repeated on eight different fractioned windows: the first min (0–1), the second min (1–2), the third min (2–3), the fourth min (3–4), the first 2 min (0–2), the last 2 min (2–4), the first 3 min (0–3), and the last 3 min (1–4). Correlation and Bland & Altman statistical analyses were systematically performed. The analysis window could be shortened to 0–2 instead of 0–4 for RMSSD only, whereas the 4-min window was necessary for LF and total power. Since there is a need for 1 min of baseline to obtain a steady signal prior the analysis window, we conclude that studies relying on RMSSD may shorten the windows to 3 min (= 1+2) in SU or seated position only and to 6 min (= 1+2 min SU plus 1+2 min ST) if there is an orthostatic test. Studies relying on time- and frequency-domain parameters need a minimum of 5 min (= 1+4) min SU or seated position only but require 10 min (= 1+4 min SU plus 1+4 min ST) for the orthostatic test.

## Introduction

Monitoring fatigue is essential for training optimization, notably for the identification of non-functional overreaching and overtraining (Meeusen et al., 2013). One of the non-invasive most commonly used tool is heart rate variability (HRV) because its changes largely depend on cardiac autonomic control, which is affected by physical training and fatigued state, for review see Buchheit (2014).

Numerous HRV test procedures have been developed to supervise athletes' follow-up: at rest either during sleep (Pichot et al., 2000; Garet et al., 2004) or awake (Schmitt et al., 2006; Plews et al., 2013), in supine and standing positions (Schmitt et al., 2013) or seated (Plews et al., 2017); with recording times varying from 1 to 15 min; during physical exercise (Sandercock and Brodie, 2006), or during the post-exercise recovery phase (Buchheit et al., 2007; Seiler et al., 2007; Hug et al., 2014). Moreover, some authors (Plews et al., 2012) rely on one HRV parameter only from the time domain: the root mean square of the successive differences (RMSSD), while others (Schmitt et al., 2016) prefer looking at multiple parameters from the time and frequency domains, which primarily are mean heart rate (mean HR), RMSSD, power in the low frequency band (LF), the high frequency band (HF), and the total power (LF+HF).

The combination of the time and frequency domains has allowed the identification of four different types of fatigue in the elite athletes, which is valuable in their training periodization (Schmitt et al., 2015b). However, the athletes have to perform a 13-min orthostatic test (7 min of recording supine followed by 6 min standing) on a very regular basis which might compromise their recovery (Schmitt et al., 2015a) or simply install a boring routine all around the year. To avoid problems of athlete compliance to complete HRV recordings, Plews et al. recommended either 5-min records (Plews et al., 2012) or recently a 1-min recording with devices as simple as photoplethysmographic (PPG) data captured at the finger level using a smartphone (Plews et al., 2017). Indeed, for RMSSD only, a 1-min window has been proposed as an alternative to the traditional 5-min window during rest or in post-exercise recovery conditions (Esco and Flatt, 2014; Pereira et al., 2016). However, none of the previous studies reported the impact of selecting a recording window as short as 1 min, on the reliability of LF, HF, and total power measurements.

With such variety in procedures, it remains particularly unclear how long a HRV recording should be in order to offer the best compromise between the quality and accuracy of the recording and the comfort for the athlete. The longer the recording time the more reliable the HRV parameters, the shorter the recording the more convenient for the athletes, especially when the recordings need to be performed on a regular basis (Plews et al., 2013). The aim of the present work is to assess the minimal acceptable recording duration at rest in the supine and the standing positions for mean HR, RMSSD, LF, HF, and total power; that is the duration below which there is a significant bias in the aforementioned HRV parameters, compared to the traditional window duration of several minutes.

## Materials and methods

The RR-interval recordings of 159 orthostatic tests from three elite athletes (53 tests each) have been gathered (Schmitt et al., 2015b). Subjects 1 and 3 are males and subjects 2 is female. Each of the three athletes has won between one and four Olympic medals, either in swimming or biathlon, plus several other titles in international and national championships. The tests selected for the work represent a follow-up between four (subject 1) and 11 (subject 3) years for each athlete. The detailed procedure of the orthostatic test can be found in details elsewhere (Schmitt et al., 2013). Briefly, the orthostatic test relied on a 13-min RR-interval recording at rest with 7 min supine (SU) followed by 6 min standing (ST). The procedures were approved by the Necker Hospital Ethic Committee (Paris, France). All the subjects provided written, voluntary, informed consent. The data analyses are based on the RR-intervals between the 3rd and 7th min SU, and between the 9th and 13th min ST. Inside those two 4-min windows, the HRV analyses were repeated on the entire 4 min (0–4) as the reference analyses and then on eight different fractioned windows: the first min (0–1), the second min (1–2) the third min (2–3), the fourth min (3–4), the first 2 min (0–2), the last 2 min (2–4), the first 3 min (0–3), and the last 3 min (1–4). Analyses were performed separately for SU and ST. Measurement of the interval duration between two R-waves of the cardiac electrical activity was performed with a HR monitor (T6, Suunto®, Vantaa, Finland).

Heartbeats that are not originated from the sino-atrial node have been shown to have drastic effects on the outcome of HRV indexes (1996). To this end, the RR-intervals from the orthostatic tests were first analyzed to remove ectopic beats from the recordings using automatic and visual inspections of the RR series. Ectopic beats were then compensated by means of interpolation to calculate normal to normal (NN) intervals. From the NN-intervals, HRV parameters were extracted namely: mean HR, RMSSD, LF (0.04–0.15 Hz) HF, (0.15–0.40 Hz), and total power (LF + HF) in ms^2^ (Schmitt et al., 2015b). The spectral power was estimated using the Fast Fourier Transform on the resampled NN-intervals (4 Hz; Vesin et al., 2016). All procedures were carried out in agreement with the Task Force recommendations (1996).

The statistical analyses include correlation and Bland & Altman (B&A) plots between the reference window (0–4) and each of the tested windows both in SU or ST. Statistical significance was set at an alpha level of 0.05. The Kolmogorov-Smirnov test was used to assess normality of the data. All the parameters presented in this work were normally distributed. All computations were performed separately for SU and ST positions using MATLAB® (MathWorks, Natick, MA, USA).

## Results

**Figure 1:**
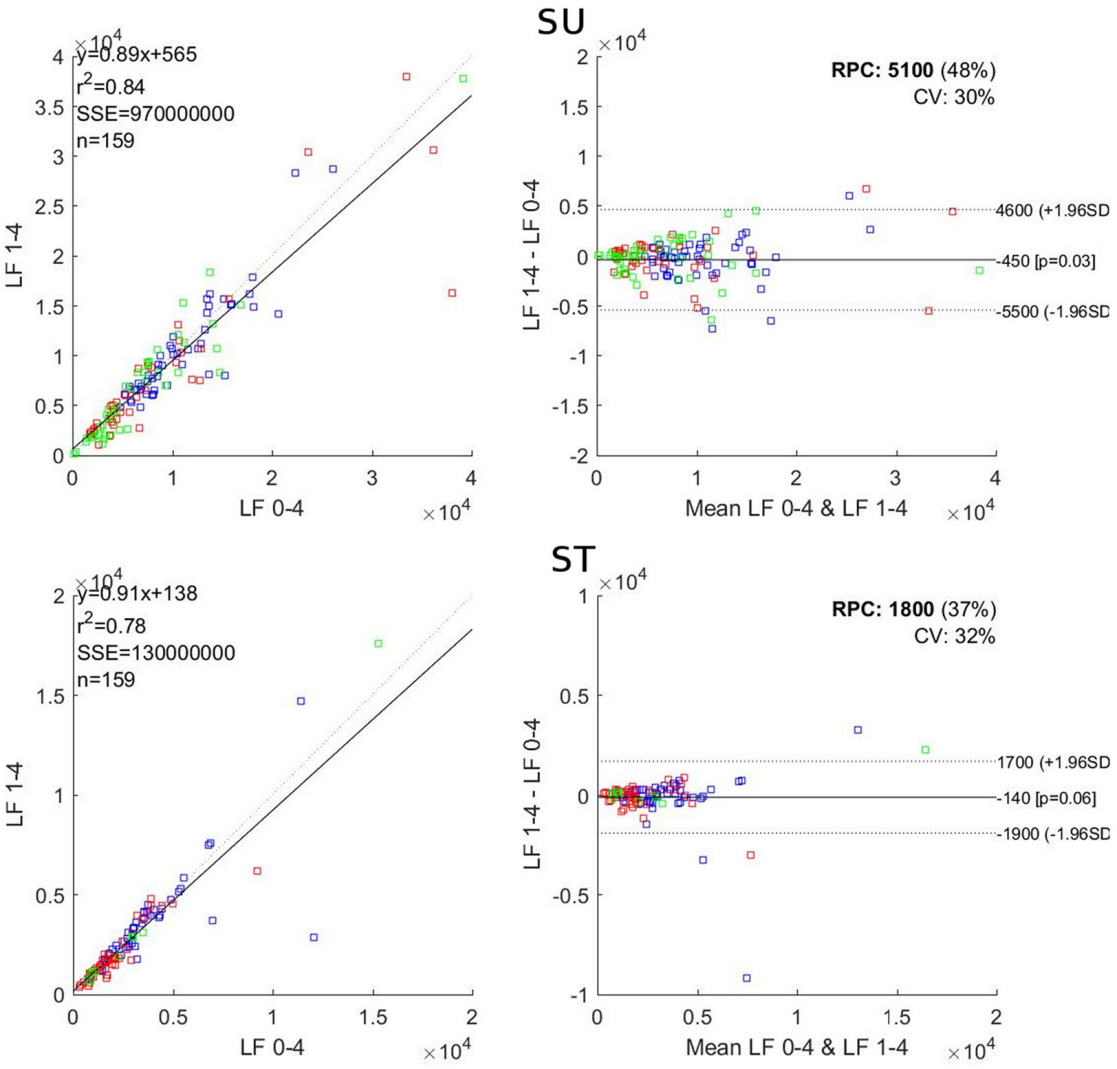
Correlation **(Left)** and Bland and Altman **(Right)** plots for comparison of LF in the 1–4 and the reference 0–4 windows. SU, supine; ST, standing; *r*^2^, Pearson r square; SSE, sum of square error; RPC, reproducibility coefficient; CV, coefficient of variation. Blue dots: Subject 1; red dots Subject 2; green dots: Subject 3.

**Figure 2:**
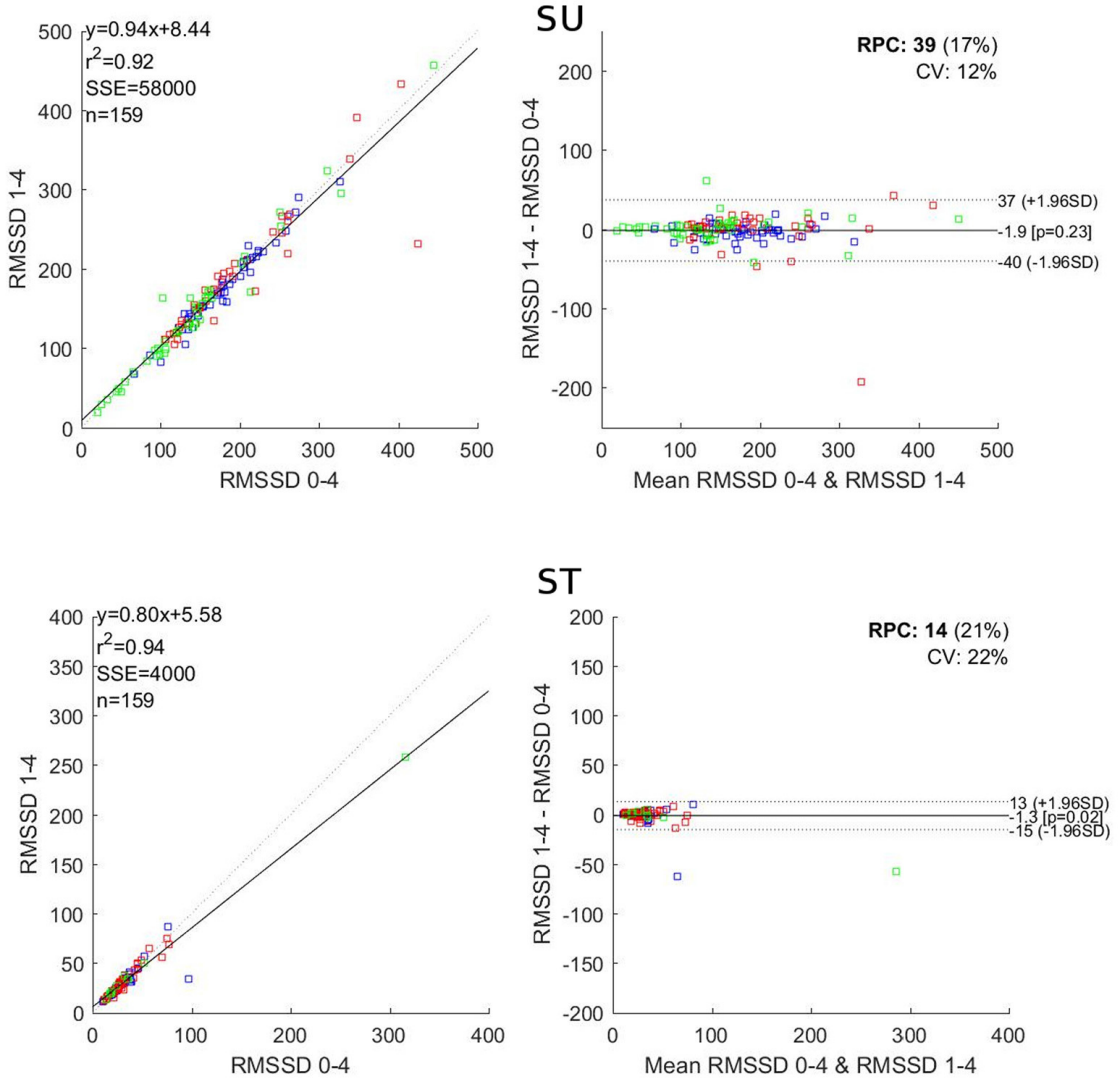
Correlation **(Left)** and Bland and Altman **(Right)** plots for comparison of RMSSD in the 1–4 and the reference 0–4 windows. SU, supine; ST, standing; *r*^2^, Pearson r square; SSE, sum of square error; RPC, reproducibility coefficient; CV, coefficient of variation. Blue dots: Subject 1; red dots Subject 2; green dots: Subject 3.

**Figure 3:**
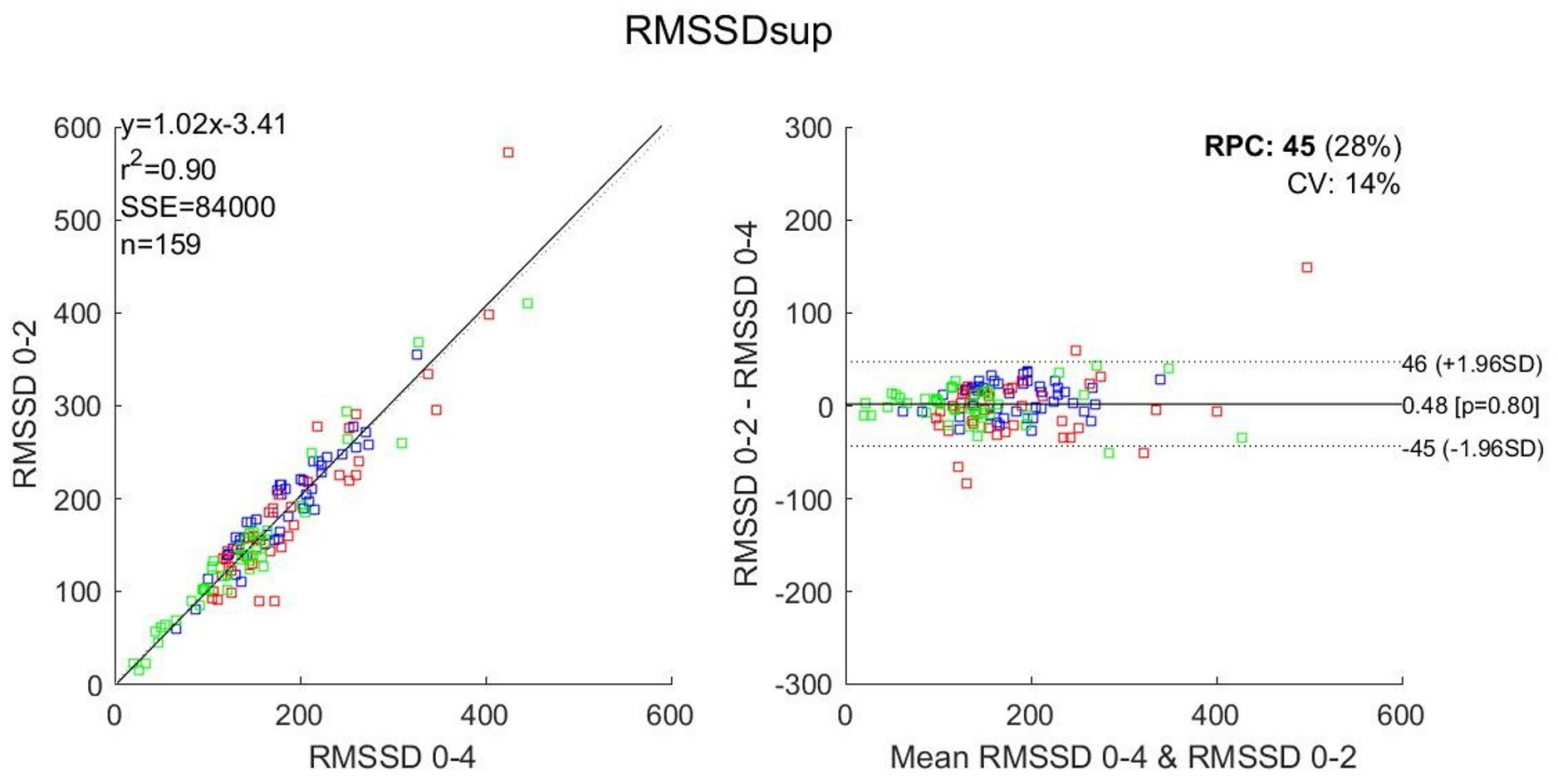
Correlation **(Left)** and Bland and Altman **(Right)** plots for comparison of RMSSD in the 0–2 and the reference 0–4 windows in the supine position. *r*^2^, Pearson r square; SSE, sum of square error; RPC, reproducibility coefficient; CV, coefficient of variation. Blue dots: Subject 1; red dots Subject 2; green dots: Subject 3.

Table 1 contains the detailed statistical data for the systematic comparison for all the tested windows against the reference window, for the correlations and the B&A. Figure 1 shows the correlation and the B&A plots for LF in SU and ST, comparing the 1–4 window to the reference 0–4 window. The 1–4 window showed the closest characteristics to the reference window for LF. Figure 2 shows the same windows for RMSSD in SU and ST. Figure 3 shows the shortest acceptable window for RMSSD (0–2) in the supine position only.

**Table 1:** Systematic correlation and Bland and Altman data for all windows comparison to the reference 0–4 window.

***HR***		**0–1**	**1–2**	**2–3**	**3–4**	**0–2**	**2–4**	**0–3**	**1–4**
*r*^2^	ST	0.66	0.75	0.73	0.78	0.81	0.90	0.88	0.97
	SU	0.57	0.81	0.59	0.71	0.85	0.78	0.94	0.86
RMSE	ST	3.80	3.37	3.65	3.73	2.74	2.14	2.05	1.06
	SU	16.80	7.14	9.96	9.52	7.17	7.21	3.76	5.66
Slope	ST	0.85	0.95	0.96	1.13	0.91	1.06	0.92	1.03
	SU	1.25	0.94	0.76	0.97	1.09	0.87	1.00	0.90
y-intercept	ST	10.43	3.62	3.12	−9.55	6.04	−4.13	5.90	−2.24
	SU	−10.30	2.56	9.23	1.88	−3.77	5.43	−0.09	4.50
Diff of Mean	ST	−1.51	−0.59	0.10	0.67	−0.80	0.44	−0.50	0.26
	SU	1.77	−0.42	−2.16	0.40	0.74	−0.56	−0.11	−0.32
*p*-value	ST	0.00	0.03	0.73	0.03	0.00	0.01	0.00	0.00
	SU	0.20	0.47	0.01	0.60	0.20	0.34	0.71	0.49
RPC (abs)	ST	8	7	7	7	5	4	4	2
	SU	34	14	21	19	14	15	7	11
RPC (%)	ST	10	9	9	8	7	5	5	3
	SU	27	18	26	24	15	20	9	16
***RMSSD***		**0–1**	**1–2**	**2–3**	**3–4**	**0–2**	**2–4**	**0–3**	**1–4**
*r*^2^	ST	0.90	0.70	0.71	0.89	0.96	0.91	0.98	0.94
	SU	0.70	0.82	0.67	0.75	0.90	0.85	0.95	0.92
RMSE	ST	11.59	9.17	11.17	9.20	5.75	6.87	3.10	5.05
	SU	45.57	30.91	42.59	37.03	23.16	26.49	15.69	19.30
Slope	ST	1.38	0.54	0.68	1.05	1.08	0.88	0.96	0.80
	SU	1.03	0.95	0.90	0.95	1.02	0.93	1.00	0.94
y-intercept	ST	−13.16	11.83	9.82	−3.12	−3.22	2.96	1.19	5.58
	SU	−3.51	2.74	5.69	9.23	−3.41	9.16	−2.07	8.44
Diff of Mean	ST	−0.18	−3.77	−1.05	−1.54	−0.35	−1.12	−0.13	−1.32
	SU	1.83	−4.98	−12.07	0.03	0.48	−2.47	−1.66	−1.88
*p*-value	ST	0.88	0.00	0.34	0.04	0.48	0.06	0.62	0.02
	SU	0.61	0.04	0.00	0.99	0.80	0.25	0.18	0.23
RPC (abs)	ST	30	29	27	18	12	15	6	14
	SU	89	61	84	73	45	53	31	39
RPC (%)	ST	56	50	44	46	33	32	19	21
	SU	41	41	51	38	28	26	20	17
***LF***		**0–1**	**1–2**	**2–3**	**3–4**	**0–2**	**2–4**	**0–3**	**1–4**
*r*^2^	ST	0.44	0.46	0.45	0.49	0.61	0.68	0.80	0.78
	SU	0.43	0.67	0.32	0.46	0.71	0.64	0.82	0.84
RMSE	ST	1,199	1,383	1,931	2,235	1,249	1,199	774	905
	SU	9,291	4,592	5,025	6,485	4,783	3,870	2,982	2,480
Slope	ST	0.56	0.67	0.91	1.15	0.82	0.92	0.82	0.91
	SU	1.24	1.02	0.54	0.93	1.17	0.80	0.98	0.89
y-intercept	ST	637	283	−61	−920	231	44	390	138
	SU	−2,677	−1,740	1,221	869	−1,695	1,107	−493	565
Diff of Mean	ST	−684	−703	−322	−483	−315	−207	−146	−138
	SU	−520	−1,596	−2,918	210	−209	−647	−664	−447
*p*-value	ST	0.00	0.00	0.04	0.01	0.00	0.03	0.03	0.06
	SU	0.49	0.00	0.00	0.68	0.59	0.05	0.01	0.03
RPC (abs)	ST	2868	2971	3786	4402	2535	2364	1654	1801
	SU	18406	8974	11425	12705	9578	7956	5831	5052
RPC (%)	ST	104	92	87	88	66	57	41	37
	SU	133	120	124	114	97	79	73	48
***HF***		**0–1**	**1–2**	**2–3**	**3–4**	**0–2**	**2–4**	**0–3**	**1–4**
*r*^2^	ST	0.81	0.82	0.19	0.89	0.88	0.93	0.88	0.96
	SU	0.46	0.78	0.57	0.68	0.79	0.78	0.90	0.85
RMSE	ST	339	328	598	620	260	266	200	189
	SU	12,525	5,859	6,015	8,033	5,991	4,860	3,318	3,853
Slope	ST	0.82	0.84	0.34	2.06	0.83	1.15	0.65	1.04
	SU	1.17	1.10	0.70	1.17	1.18	0.92	0.98	0.92
y-intercept	ST	99	57	395	−543	91	−71	177	−34
	SU	−259	−968	1,445	−660	−923	576	93	462
Diff of Mean	ST	−6	−39	9	77	−11	16	−30	−10
	SU	1299	−73	−1,294	848	715	−178	−88	−259
*p*-value	ST	0.83	0.17	0.89	0.37	0.64	0.50	0.29	0.49
	SU	0.20	0.88	0.02	0.19	0.15	0.65	0.74	0.41
RPC (abs)	ST	726	696	1,598	2,129	584	574	703	375
	SU	24,699	11,608	13,144	16,025	12,223	9,633	6,495	7,686
RPC (%)	ST	109	100	93	94	67	60	40	37
	SU	98	90	98	88	64	64	48	47
***Total p***		**0–1**	**1–2**	**2–3**	**3–4**	**0–2**	**2–4**	**0–3**	**1–4**
*r*^2^	ST	0.47	0.42	0.44	0.45	0.64	0.65	0.81	0.73
	SU	0.41	0.73	0.42	0.54	0.74	0.69	0.84	0.86
RMSE	ST	2,468	2,848	4,624	4,964	2,661	2,773	1,731	2,306
	SU	24,685	12,279	12,504	17,189	12,769	10,508	8,119	7,004
Slope	ST	0.52	0.54	0.91	0.99	0.78	0.82	0.79	0.84
	SU	1.07	1.05	0.55	0.98	1.11	0.81	0.98	0.92
y-intercept	ST	945	768	−882	−1529	361	482	891	685
	SU	−5,154	−6,469	2,449	−239	−4,072	2,373	−1,179	938
Diff of Mean	ST	−2,425	−2,462	−1,538	−1,625	-1,197	−746	−597	−429
	SU	−3,276	−5,141	−9,177	−789	−1,153	−2,469	−1,823	−1,237
*p*-value	ST	0.00	0.00	0.00	0.00	0.00	0.00	0.00	0.03
	SU	0.10	0.00	0.00	0.56	0.26	0.01	0.01	0.03
RPC (abs)	ST	6,450	6,915	9,072	9,699	5,563	5,638	3,875	4,723
	SU	48,306	24,067	29,672	33,592	25,303	21,695	15,889	14,043
RPC (%)	ST	100	92	88	88	68	60	44	39
	SU	113	96	104	99	82	71	63	42

*SU, supine; ST, standing; r2, Pearson r square; RMSE, root mean square error; RPC, reproducibility coefficient*.

For RMSSD, the 3-min windows showed *r*^2^ values ranging from 0.92 to 0.98, a mean bias between −1.88 and −0.13 and a significant difference for the 1–3 window in ST. The 2-min windows showed *r*^2^ values ranging from 0.85 to 0.96, a mean bias between −2.47 and 0.48 and showed no significant difference. The 1-min windows showed *r*^2^ values ranging from 0.67 to 0.90, a mean bias between −12.07 and 0.03 and significant differences for 1–2, 2–3, and 3–4 windows.

For LF, the 3-min windows showed *r*^2^ values ranging from 0.78 to 0.84, a mean bias between −664 and −138 and a significant difference for the 1–3 window in ST. The 2-min windows showed *r*^2^ values ranging from 0.61 to 0.71, a mean bias between −647 and −207 and showed significant difference for the 0–2 window in SU. The 1-min windows showed *r*^2^ values ranging from 0.32 to 0.67, a mean bias between −2,918 and 210 and significant differences for 0–1 and 3–4 windows.

For HF, the 3-min windows showed *r*^2^ values ranging from 0.85 to 0.96, a mean bias between −259 and −30 and showed no significant difference. The 2-min windows showed *r*^2^ values ranging from 0.78 to 0.93, a mean bias between −178 and 715 and no significant difference. The 1-min windows showed *r*^2^ values ranging from 0.19 to 0.89, a mean bias between −1,294 and 1,299 and a significant difference for the 2–3 window in SU.

For total power, the 3-min windows showed *r*^2^ values ranging from 0.73 to 0.86, a mean bias between −1,823 and −429 and significant difference in all cases. The 2-min windows showed *r*^2^ values ranging from 0.64 to 0.74, a mean bias between −2,469 and −597 and significant differences for all cases except one. The 1-min windows showed *r*^2^ values ranging from 0.41 to 0.73, a mean bias between −9,177 and −789 and significant differences in all cases except two.

## Discussion

In this work, we present a systematic comparison between an extended version of the orthostatic test, 13 min in total resulting in 4-min long analysis windows, and shortened analysis windows. Our main findings are that the analysis windows, when shortened, become less reliable in a significant manner for LF and total power. However, regarding RMSSD, shortening the analysis windows from 4 to 2 min is acceptable. In all regards 1-min windows led to deteriorated results.

The parameter most sensitive to window time reduction was LF. Cutting the first min of the 4-min reference window induced significant differences between the two windows (*p* < 0.05 SU; *p* ≈ 0.06 ST) and diminished the correlation coefficient (*r*^2^ = 0.84 SU; *r*^2^ = 0.78 ST) as seen in Figure 1. Yet, the 1–4 window should be the closest possible to the reference window as the subjects have been resting in a quiet environment, in supine position, for the last 4 min before the start point of that particular window. The LF band encompasses frequencies as low as 0.04 Hz, which means that, in 4 min, there is a maximum of only 9.6 full waveforms, which drops at 7.2 for 3 min. This drop appears sufficient to significantly alter the LF value when the window is shortened by 1 min. In the HF band the lowest selected frequency is 0.15 Hz, which means that over a 4-min window there is a maximum of 36 full waveforms, which drops at 27 for 3 min, a number still sufficient to keep good consistency between 3 and 4-min windows (*r*^2^ above 0.85 and no significant difference between the windows). The total power is negatively affected (poor *r*^2^ and significant differences both SU and ST) because LF is affected. Al Haddad et al. (2011) showed that the frequency domain parameters were less reproducible than the time domain ones during post-exercise recovery; which is in favor of an impaired LF when shortening the recording window.

A previously published study accurately categorizes the individual patterns of five HRV parameters in “no fatigue” and four types of fatigue (Schmitt et al., 2015b). These distinct patterns encompass increases and/or decreases in HR as well as in LF and HF components. These changes are differently sized in supine and standing positions, and sometimes directed contrariwise in each position. A main outcome of this previous work was that supine and standing HRV variables were fully independent, and non-commutable in the clustering of alterations from the individual normal “no fatigue” patterns (Schmitt et al., 2015a). So, LF is a major element of the HRV analysis and should be included on a regular basis, which makes the 4-min window mandatory. In the context of the entire orthostatic test this corresponds to 1 min resting supine followed by 4 min of recording plus 1 min standing followed by 4 min of recording, thus a total of 1+4+1+4 = 10 min.

However, we acknowledge that RMSSD measures are commonly recommended and therefore might have an effective practical usefulness to help the practitioner to identify a global “fatigue” level. In the perspective of reducing the duration of the orthostatic test to the drastic minimum, only using RMSSD, a 2-min window should be used; which in the context of the entire orthostatic test would make 1 min resting supine followed by 2 min of recording plus 1 min standing followed by 2 min of recording, thus a total of 1+2+1+2 = 6 min. Reducing the duration of the HRV data collection any further implies the suppression of the standing position, which would result in the supine only 1+2 = 3 min HRV test, as illustrated in Figure 3. Any test shorter than 3 min is dangerously exposed to imprecise computation of both the temporal and frequency domains which may result in the wrong diagnosis of the “fatigue” or “no fatigue” state. Accordingly, Munoz et al. (2015) studying a cohort of 3,387 adults, concluded that RMSSD should not be assessed using recordings shorter than 2 min, which is in accordance with the present work, focusing on elite athletes.

Esco and Flatt (2014) proposed to reduce the recording windows to 1 min for RMSSD, despite significant differences vs. their reference 5-min window. Pereira et al. (2016) also found that a 1-min window was acceptable for RMSSD assessment, however their correlation coefficient was 0.85 (which for us is the edge of the acceptability). Moreover, they did not report whether the 1-min window induced significant difference when compared to the 5-min reference window. Additionally, when assessing the seasonal changes in futsal players, these authors relied on 5-min recordings rather than 1-min (Oliveira et al., 2013). In these studies as in most previous ones (Nakamura et al., 2017), the tested populations were very homogeneous, athletes, usually from a single team, taken at a given moment of their sport season; which is in favor a good reliability when reducing the recording duration, but which is clearly not representative of the intra-individual variations of the HRV parameters over longer periods (e.g., a whole year), potentially including several changes in fitness and fatigue states. In the present dataset, there are recordings corresponding to fatigue and non-fatigue states that were collected at various moments of the season, therefore corresponding to various training and competing modalities, including seasonal changes. In other words, our dataset encompasses a multitude of athletes' autonomic responses corresponding to an actual follow-up over several years and shows that under these circumstances the reliability of the 1-min window duration is questionable. Additionally, previous studies providing daily exercise prescription based on HRV over several weeks of follow-up also use windows of several minutes to assess the time domain parameters (Kiviniemi et al., 2010).

Several studies showed good reliability of ultra-short HRV recordings (<1 min) considering RMSSD. However, most of them focused on the post-exercise recovery phase (Goldberger et al., 2006; Al Haddad et al., 2011; Esco and Flatt, 2014; Gomes et al., 2017) which is not the purpose of the present study. Munoz et al. (2015) claimed that 10 s recordings, showing a *r*^2^ value of 0.728 compared to 3-min recordings is “substantial,” yet concluded that RMSSD should not be assessed using recordings lasting less than 2 min. To our knowledge, 10, 20, or 30 s recording windows have never been proposed as a procedure to ensure athletes' follow-up.

The European and American societies concerned (1996) recommend that the recording should last for at least 10 times the wavelength of the lower frequency bound of the investigated component, which is the case for our 4-min window encompassing 9.6 waveforms for the LF band. The societies also stated that the LF band requires the most extended recording duration with regard to the time domain parameters, which is in accordance with our present findings. Finally, the societies recommend 5-min recordings of a stationary system. The present work indicates that the last 4 min are sufficient for the window analysis, the first min being dedicated to the reaching of the stationary state of the subject.

In the orthostatic test originally designed by Schmitt et al. (2013), there are 3 min supine before the analysis window starts. This period is dedicated to let the respiratory rhythm settle to a basal rest level so that the respiratory sinus arrhythmia (RSA) does not drift during the HRV test, possibly jumping from HF to LF (and vice-versa) and consequently inducing misinterpretation of the frequency domain parameters (Saboul et al., 2014; Wessel et al., 2016). Although it is essential that the athletes be in a complete resting state when performing the HRV test (ideally in the morning upon waking up), we believe that reducing the time period before the start of the analysis window to 1 min is sufficient. However, future research is necessary to confirm the minimal duration needed. Also, future research should focus on the development of a tool for the extraction of the respiratory rate without a reference respiration signal (Mirmohamadsadeghi et al., 2016).

## Conclusion

The present work shows that reducing the window duration for HRV analysis below 4 min negatively affects the outcomes for the low frequency band and the total power. Additionally, we found that reducing the window duration to 2 min is acceptable when RMSSD only is considered. 1-min windows significantly deteriorate the HRV analysis for all parameters. When both the time and the frequency domain parameters are considered we recommend 5 min supine followed by 5 min standing, the analyses being performed on the last 4-min in each position resulting in a 10-min orthostatic test. When RMSSD only is considered, 3 min supine seems the minimal duration or 3 min supine followed by 3 min standing should be sufficient, resulting in a 6-min orthostatic test.

## Author contributions

LS and GM designed the study; LS collected the data; NB, SY, and JV performed the signal processing and data analysis; NB drafted and wrote the article; NB prepared the figures and performed the statistical analysis; LS, SY, JV, and GM reviewed the manuscript. All authors approved the final version of the manuscript.

### Conflict of interest statement

The authors declare that the research was conducted in the absence of any commercial or financial relationships that could be construed as a potential conflict of interest.
